# Automatic adjustment of ventricular antitachycardia pacing and individualized device therapy

**DOI:** 10.1016/j.hrcr.2021.11.026

**Published:** 2021-12-06

**Authors:** Rajiv Tripathi, Christopher Gubran, Craig Jeavons

**Affiliations:** ∗ Gloucestershire Royal Hospital, Gloucester, United Kingdom; † Medtronic, Newport, South Wales, United Kingdom

**Keywords:** Pacing, Defibrillator, ATP, iATP, Antitachycardia pacing algorithm, Medtronic, CRT-D, Ventricular tachycardia

**Key Teaching Points**

• Antitachycardia pacing (ATP) is a safe, pain-free, and effective method of terminating ventricular tachycardias.
• There are several different algorithms of ATP; the most frequently programmed are burst and ramp. Burst pacing is a train of impulses with equal interstimulus intervals. Ramp is a train of impulses with decrementing interstimulus intervals.
• While ATP is an important tool in the arsenal of treating ventricular arrhythmia, it may not work for all tachycardias; consider the substrate being treated as well.
• In some cases, such as arrhythmogenic right ventricular dysplasia, ramp pacing was found to be much less effective than burst pacing in terminating tachycardia as well as having a higher probability of acceleration.
• Intrinsic ATP is a new algorithm present in Medtronic defibrillators that allows for the delivery of patient- and arrhythmia-specific therapy by using calculations based on the tachycardia cycle length and origin to determine the most accurate pacing timing to terminate the tachycardia.

## Introduction

Intrinsic ATP™ (iATP™) is an automated ventricular antitachycardia pacing (ATP) algorithm that provides individualized therapy. Each ATP attempt, if unsuccessful, is sequentially adjusted based on the postpacing interval. This algorithm was tested and proven to be beneficial in virtual modeling studies and patient cohorts. Here we describe a case where manually programmed attempts with conventional ATP failed to terminate a slow incessant ventricular tachycardia (VT) but iATP proved clinically more efficacious.

## Case report

**Figure 1:**
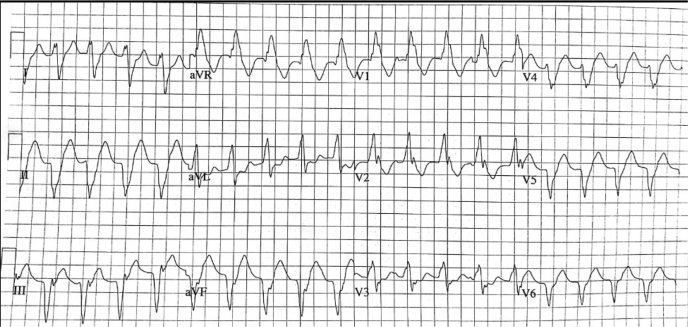
Electrocardiogram showing ventricular tachycardia at a rate of 120 beats per minute.

An 81-year-old man was admitted to our coronary care unit with light-headedness and palpitations coincident with episodes of slow VT (Figure 1). He had a background history of coronary artery bypass grafting in 1997 with 3 venous grafts, severe left ventricular (LV) systolic dysfunction, and paroxysmal atrial flutter.

On clinical examination he had a blood pressure of 124/81, pulse of 128 beats per minute (bpm). His oxygen saturation was 98% on air. He was alert and responsive and appeared comfortable at rest. Median sternotomy and saphenous venous harvest site scars were well healed. Cardiovascular and respiratory examinations were otherwise unremarkable.

Initial work-up showed normal renal function, electrolytes, and normal full blood count. A chest radiograph revealed globular cardiomegaly, without pulmonary congestion. Electrocardiograms were consistent with VT and an inferobasal LV exit site given the remote history of inferior infarction and the QRS morphology. High-sensitivity troponin T was mildly elevated (23.5 ng/L). NT-proBNP was not assayed.

Intravenous amiodarone was infused at 300 mg over 1 hour followed by 900 mg over 24 hours. DC cardioversion was performed and an externalized dual-chamber pacemaker was implanted to manage intermittent complete AV block. The pacemaker was programmed DDI at 80/min and paced AV delay of 300 ms. After 2 days of amiodarone loading, the incessant VT settled and the patient was 100% atrially and ventricularly paced.

**Supplemental Figure 1:**
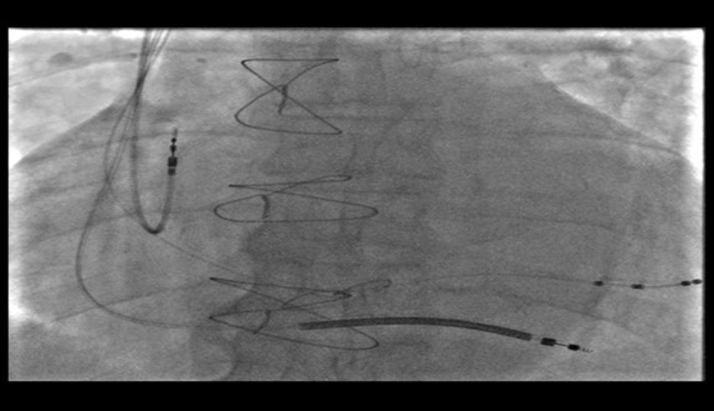
Fluoroscopic image taken at the end of implant procedure demonstrating lead positions in the PA view.

Transthoracic echocardiography demonstrated severe LV systolic dysfunction with LV ejection fraction 25%–30%; LV outflow tract velocity time interval was 14.2 cm and stroke volume was calculated as 59 mL with a cardiac output of 3.5 L/min. The end-diastolic volume was 157 mL. His inferior segments were thinned and akinetic with severely hypokinetic inferoseptal and inferolateral regions and otherwise global hypokinesis. The right ventricle (RV) was dilated with reduced longitudinal function and a hypokinetic RV apex. There was moderate, functional tricuspid regurgitation. Coronary angiography demonstrated occluded venous grafts, a chronic total occlusion of the right coronary artery, and severe left anterior descending artery disease. Percutaneous coronary intervention to the left anterior descending artery was performed. His chronic total occlusion of the right coronary artery was not revascularized, given the likelihood of nonviability. A subpectoral cardiac resynchronization defibrillator (CRT-D) was implanted (Supplemental Figure 1). Ventricular fibrillation (VF) induction and defibrillation threshold testing were not performed in view of suspended anticoagulation and recent atrial flutter. He was programmed AAI to DDD with a base rate of 50 bpm. He was set for backup CRT, as his intrinsic QRS was narrow with a duration of 106 ms when not in AV block. His initial tachycardia programming was a single treatment zone for “VF” at rate >188 bpm, 30/40 with 3 sequences of iATP prior to and during charging, followed by full-output (40 J) shocks (6). A monitor zone was programmed at 102 bpm with 130 beats needed for detection, as the VT was well tolerated, and recurrence was to be checked at follow-up. Prior to discharge he remained hemodynamically stable but unfortunately was unable to tolerate amiodarone. He developed an intractable and disabling tremor and was subsequently discharged on bisoprolol 10 mg daily.

Two weeks after discharge, his VT recurred after amiodarone cessation and, as this was untreated by the device (in monitor zone) manual, ATP was attempted. The VT was unaffected by multiple sequences of traditional ventricular ATP (burst and ramp ATP with various numbers of pulses, coupling intervals, and R-R% adjustments). ATP was delivered by RV pacing. The most aggressive manual ATP attempted was a Ramp sequence of 12 pulses at 78% R-R interval and decrementing by 30 ms; minimum interval 150 ms.

**Figure 2:**
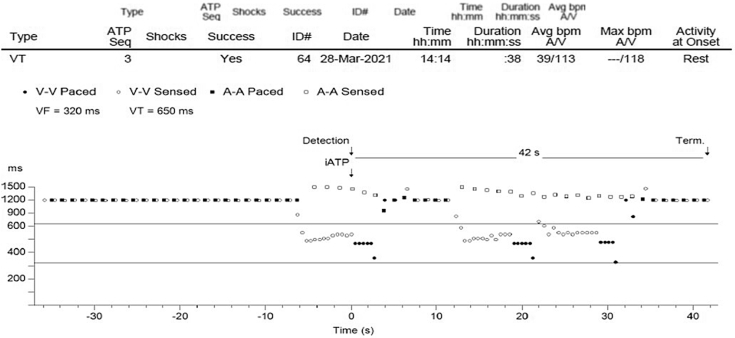
Image taken from Medtronic programmer demonstrating successful use of the Intrinsic ATP (iATP) automated ventricular antitachycardia pacing algorithm. There were three sequences of iATP delivered, which eventually led to termination of the tachycardia. There was a termination of the ventricular tachycardia (VT) by the first attempt, and it restarted prior to meeting the episode termination rule (8 consecutive R-Rs outside enabled therapy zones, not counting the postpacing interval [PPI] and the one after it as the redetection algorithm initializes on that event); there were 7 events, then an event in the VT zone. Because the PPI was long (paced at 1200 ms; longest possible cycle), the closed-loop analysis is “Break,” so if there is a redetection and no change in the VT, iATP will not decrement S2 on the next sequence. If S2 continued to decrement, eventually it would lose capture and stop working—or increase acceleration risk. This is unique to iATP. In this case the non-reset limit was 1035 ms, making the break lower limit 1135 ms, so the PPI of 1200 was called a break (correctly); the redetected VT cycle length was within 10% of the prior, so S2 was not decremented.

**Figure 3:**
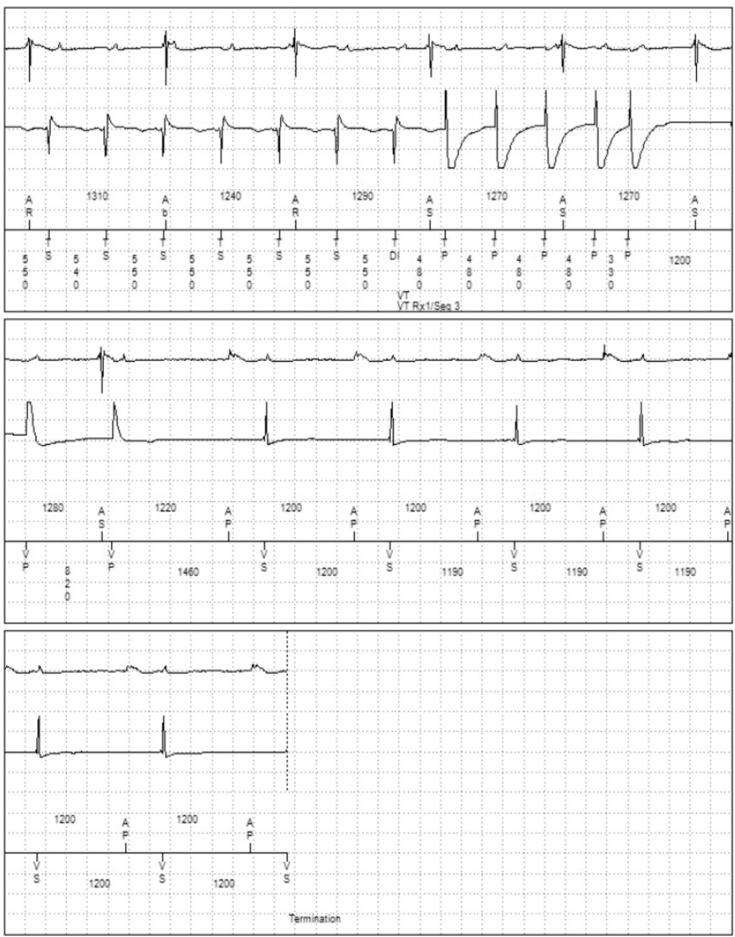
Image taken from Medtronic programmer demonstrating the successful conversion of ventricular tachycardia to sinus rhythm owing to the Intrinsic ATP (iATP) automated ventricular antitachycardia pacing algorithm. There are 2 ventricular paced beats after conversion, then intrinsic rhythm takes over (as per device programming).

**Supplemental Figure 2:**
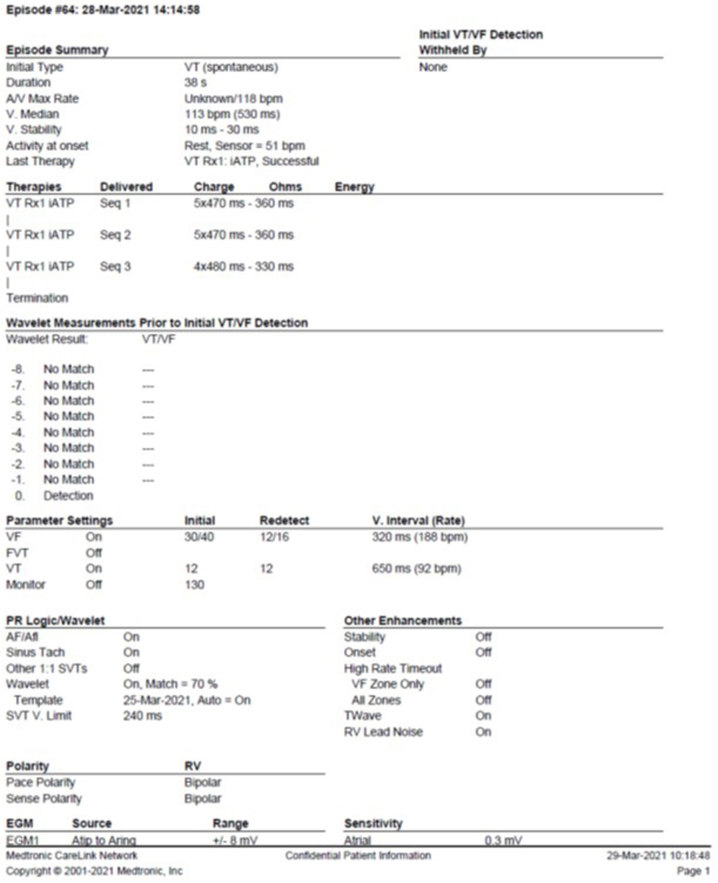
Episode summary as documented via a Medtronic programmer showing summary of tachycardia and subsequent therapies thereafter that led to restoration of sinus rhythm.

Programming of iATP, a novel company-specific algorithm, following the above failed attempts proved successful at terminating the incessant VT at the first attempt. One minute later, however, the VT reinitiated. He was left in sustained, well-tolerated VT for a total of 2 weeks. The patient was started on sotalol and up-titrated to a dose of 160 mg twice a day. Following this, with iATP reactivated (Figure 2), the VT was pace terminated by the device, with sustained restoration of atrially paced, ventricularly sensed rhythm (Figure 3 and Supplemental Figure 2). He remained stable, with intermittent recurrence of VT that was managed with iATP treatment; there were 7 total further episodes of VT treated with iATP (at most 3 cycles per episode) in the month that followed. He was then seen by an electrophysiology team at a tertiary center who opted to continue monitoring in preference to a VT ablation owing to how well the algorithm worked and how clinically asymptomatic he remained. No episodes of acceleration to faster VT cycle length or degeneration to VF have occurred in this gentleman. He is clinically stable currently and routinely followed up at the heart failure and complex device clinic.

## Discussion

ATP is a mode of treatment offered for termination of VT by intracardiac defibrillators. ATP allows for painless treatment of VT with less battery consumption and possibly less detrimental effects on the myocardium.^1^ It involves bursts of pacing, usually 5–15 pulses, with the goal of penetrating and interrupting the VT circuit. Studies have shown ATP capable of terminating up to 90% of VTs with a cycle length of >300 ms (rate of <200 bpm).^2^ There is a risk of acceleration, which is defined as a 10% or greater decrease in the VT cycle length after ATP or degeneration to VF; these may occur in up to 10% of ATP therapy. The modes of traditional ATP are either burst or ramp protocols. Burst pacing is a train of impulses with equal interstimulus intervals. Ramp is a train of impulses with decrementing interstimulus intervals.

Studies have compared burst vs ramp overdrive pacing for both slow (<188 bpm) and fast VT. Overall, there is not a clear difference in the efficacy of burst or ramp ATP, in ischemic and nonischemic cardiomyopathies.^3^ In most cases, the choice of programming is based on clinician’s preference or manufacturer nominal settings. Ramp sequences may have a greater likelihood of acceleration, especially in some patient cohorts, for example arrhythmogenic cardiomyopathy. Nonprogramming factors that play a role in the effectiveness of ATP include the lead position and its proximity to the tachycardia circuit (septal vs apical lead positions related to individual tachycardia exit sites), as well as the biochemical status of the patient (electrolytes, pH), medications (antiarrhythmic drugs), and acute ischemic events. Owing to the array of confounders, it is exceedingly difficult to tailor ATP specifically to each patient and, as such, most patients receive empiric programming, not necessarily specific to their underlying conditions.^4^

The alterations that can be made to the standard ATP algorithms include the following: (1) Changing the coupling cycle, defined as the delay between the last sensed and the first paced ventricular event. The shorter the coupling interval, the higher the chance of accelerating the tachycardia. (2) Changing the duration and/or number of sequences of ATP or the amplitude of the pulses. (3) Changing the site of the stimulus, only possible in CRT devices that can deliver ATP via the LV lead. (4) Device manufacturer–specific sequences, such as Sweep pacing in Boston Scientific devices, Burst + from Biotronik, and the new iATP algorithm from Medtronic.^5^

iATP is a novel Medtronic ICD algorithm that offers specific and individualized ATP therapy that may be more efficacious in some cases. iATP delivers patient- and arrhythmia-specific therapy by using calculations based on the tachycardia cycle length and origin to determine the most accurate pacing timing to terminate the tachycardia.

The iATP algorithm involves automated ATP where each sequence “learns” from the prior sequence and alters the therapy. The algorithm designs initial ATP sequences based on recent heart rate data. Subsequent sequences are based on analysis of the postpacing interval of the prior ATP sequence, to determine whether or not the train of impulses reached the VT circuit, or lost capture. The iATP S1 coupling interval is set to 88% of the VT cycle length. The number of S1s is computed from pacing electrode–to–VT circuit travel time, assuming a long travel time for initial sequences. If reset is not achieved, additional S1 pulses are subsequently added. If the initial sequence achieved reset, iATP estimates actual travel time and minimizes the number of S1 pulses in subsequent bursts to the number needed for reset.

The S2 impulse is intended to advance VT circuit timing and close the excitable gap. In contrast to the longer S1 coupling interval (88%VT cycle length), the S1-S2 coupling is intended to be as short as possible without loss of capture. Using heart rate data analysis, the initial S2 is delivered just beyond the predicted myocardial effective refractory period, and decremented by 20-30 ms with each unsuccessful attempt, provided capture is achieved. Once loss of capture occurs, the S2 is restored to the shortest coupling interval that captured the myocardium. An S3 pulse is then added, and S3 pulse is decremented thereafter to continue searching for efficacy until minimal coupling of 160 ms has been attempted. By this time, if shocks are programmed, shock therapy would be delivered. If criteria for adjustment of the clinical arrhythmia (>10% change in VT cycle length) are met at any time during iATP therapy, the algorithm is reset to the initial S1 impulse determination stage.

iATP was tested in a clinical setting in patients who had DR-implantable cardioverter-defibrillators (ICDs) or CRT-ICDs, a history of ≥1 ICD-treated VT/VF episode, or sustained monomorphic VT. A total of 669 sustained monomorphic VTs from 49 patients were adjudicated, with an overall termination rate of 80.1%.^6^

iATP was also tested in a virtual scenario using magnetic resonance imaging and electrophysiology data via CARPEntry simulation. Reentrant VT was generated with 259 unique ATP scenarios from 7 scarred hearts. In this study iATP was found to be 17% more effective than traditional ATP in terminating tachycardia without increasing the rate of acceleration. This suggested that iATP may be more successful with complex scar-related tachycardias.^7^

In addition to patient and arrhythmia specificity, iATP is easier to program, with less room for error from pacing physicians or physiologists.

One form of treatment not explored in this case was that of low-energy shocks (<2 J). Generally, when programming low-energy shocks,^8^ it counts toward total shocks delivered and can therefore limit the amount of high-energy shocks that can be delivered in sequence.^9^ This, coupled with the risk of acceleration and ventricular fibrillation, limits the use of low-energy shocks; however, it would have been considered in the absence of successful treatment by iATP.

## Conclusion

This case demonstrates a real-world example of Medtronic’s iATP algorithm successfully terminating incessant slow VT that was otherwise refractory to both pharmacological and traditional ATP therapy.
